# Construction Notes

**Published:** 1893-09-30

**Authors:** 


### CONSTRUCTION NOTES.

### Foresters' Convalescent Home, Clent.

The foundation-stone of this institute has recently been laid. The old home which this newer one has superseded provided accommodation for 13 patients only, whilst this, the natural outcome of the former, will accommodate 30 patients. Site and building will entail a cost of £2,500, and the members of the district federated in the cause have raised £1,000 towards the required sum. The designs were entrusted to Mr. B. Corser, of Birmingham, and the contractors are Messrs. Gleest and Son, of Brettell Lane. In style the building is of a Gothic character, red bricks, with stone facings, being employed in this construction. A verandah runs along the front. On the ground floor is a large dining-room, day room, and the necessary domestic rooms and offices. On the floor above these will be dormitories for the patients and the rooms for attendants. In laying out the building, the architect has so arranged that the home admits of further extension should this step be felt necessary at a later period.

### The Corbett Hospital, Stourbridge.

The formal opening of this Hospital took place on July 31st. The approach to the building is by a fine avenue of elm trees. The Hospital itself contains five wards, a men's medical ward containing six beds, a woman's medical ward containing four beds, two private wards (one for two beds and the other for one), and an accident ward with six beds. This makes a total of eighteen beds altogether, and the whole of the wards are on the ground floor. The male and female medical wards, the female special ward, the nurses' room, and the male special ward are to the right on entering the main door of the Hospital, while to the left are committee-room, Surgeon's consulting-room, dispensary, operating-room, store-room, kitchen, larder, and scullery. Here are also bath-rooms, lavatories, *post-mortem* room, &c., and a mortuary provided, the latter trio being separate erections adjoining the main building. Special attention has been directed to the ventilation throughout. Hot-water apparatus is supplied with radiators fixed in each of the corridors and wards. An excellent supply of water has been obtained by laying large sized pipes from the main to the hospital, and fire hydrants have been fixed in case of emergency. The building is a three-storey one, and on the second floor are to be found the rooms set aside for meetings of the Board of Governors, the matron's room, and the other rooms for the accommodation of the staff. The drainage has been carried out with all the latest sanitary improvements. The architect designing this hospital was Mr. T. Robinson, whilst Mr. W. North contracted for the building of it, and Messrs. Jones and Attwood supplied the heating apparatus.

